# Insight into the role of phosphatidylserine in complement-mediated synapse loss in Alzheimer’s disease

**DOI:** 10.12703/r/10-19

**Published:** 2021-02-24

**Authors:** Dimitra Sokolova, Thomas Childs, Soyon Hong

**Affiliations:** 1 UK Dementia Research Institute, Institute of Neurology, University College London, Gower Street, London WC1E 6BT, UK

**Keywords:** Alzheimer’s disease, mitochondrial dysfunction, synapse loss, classical complement cascade, microglia, astrocyte, phosphatidylserine, synaptosis, caspase-3, MFG-E8, TAM, TREM2

## Abstract

The innate immune system plays an integral role in the brain. Synaptic pruning, a fundamental process in developmental circuit refinement, is partially mediated by neuroimmune signalling at the synapse. In particular, microglia, the major tissue-resident macrophages of the brain, and the classical complement cascade, an innate immune pathway that aids in the clearance of unwanted material, have been implicated in mediating synapse elimination. Emerging data suggest that improper signalling of the innate immune pathway at the synapse leads to pathological synapse loss in age-related neurodegenerative diseases, including Alzheimer’s disease. Now the key questions are whether synapses are targeted by complement and, if so, which synapses are vulnerable to elimination. Here, we review recent work implicating C1q, the initiator of the classical complement cascade, and surrounding glia as mediators of synapse loss. We examine how synapses could undergo apoptosis-like pathways in the Alzheimer brain, which may lead to the externalisation of phosphatidylserine on synapses. Finally, we discuss potential roles for microglia and astrocytes in this ‘synaptic apoptosis’. Critical insight into neuroimmune regulatory pathways on synapses will be key to developing effective targets against pathological synapse loss in dementia.

## Introduction

Genetic studies in Alzheimer’s disease (AD) implicate microglia, the major resident immune cells of the brain, as modulators for the risk of dementia^1–5^. Studies in animal models of AD suggest that microglia may contribute to the risk by acting as cellular mediators of synapse loss^6–11^. One proposed mechanism for the microglia-mediated synapse loss involves a region-specific reactivation of an innate immune pathway called the classical complement cascade, which has been shown to play a critical role in developmental synaptic pruning^12,13^. However, what triggers this reactivation of the complement-mediated synapse pruning pathway is unclear. In particular, how synapses may be lost in AD is a critical question that needs to be elucidated. Literature in AD models suggests an interesting concept of ‘synaptosis’, whereby focal apoptotic cascades at dendritic spines can occur in the absence of neuronal death^14–16^. This raises the intriguing questions of whether complement-mediated synapse loss by microglia in AD requires synaptosis and, if so, how. Here, we summarise emerging data from developing and diseased brains which suggest a role for phosphatidylserine (PtdSer), a canonical ‘eat me’ signal on apoptotic cells, in synapse loss. We then discuss potential links between externalised phosphatidylserine (ePtdSer), complement (C1q and C3) and receptors on microglia and astrocytes that could be involved in the recognition of ePtdSer. Furthermore, we speculate on whether and how ePtdSer may act as a signal for synaptosis in the AD brain. Synapse loss is a significant correlate of cognitive impairment in AD^17–22^. Therefore, critical insight into mechanisms mediating synapse loss has the potential to identify effective therapeutic targets against cognitive decline and alter AD prognosis.

## Complement-mediated synapse loss

A universal hallmark of neurologic diseases is the region-specific vulnerability of neurons and neuronal networks to dysfunction and loss^23^. Hence, a long-standing question in neurobiology has been what contributes to the region-specific loss of synapses and neurons. In AD, synapse loss strongly correlates with cognitive impairment^17–22^ and appears to be present before overt neuronal loss^24,25^. Data from multiple laboratories collectively suggest that synaptic failure and loss in AD are likely initiated by pre-fibrillar oligomers of amyloid-beta (Aβ) and tau at synapses^6,26–33^. However, precise mechanisms of how these oligomers initiate synapse loss and dysfunction need further investigation.

Insight into the role of the innate immune pathway in synapse loss stemmed from post-natal circuit refinement in the developing mouse brain. Synaptic pruning in the developing brain is a normal and highly regulated process, where supernumerary synapses are removed to obtain the appropriate number of synapses^34^. Multiple mechanisms have been shown to mediate synaptic refinement in the developing brain, depending on brain regions and timepoints^35,36^. These include immune pathways such as fractalkine signalling^37,38^ and triggering receptor expressed on myeloid cells 2 (TREM2)^39^ in the hippocampus, complement (C1q/C3)^12,13^, MERTK-MEGF10^40^ and IL-33^41^ in the visual thalamus, MHC class I-PirB^42–45^ in the visual cortex, and fractalkine signalling and ADAM10^46^ in the barrel cortex. Among these, the classical complement cascade (C1q and C3) has been shown to be reactivated in multiple models of neurologic diseases^6,8–10,47–51^. Complement proteins are innate immune molecules that act as ‘eat me’ signals to promote rapid clearance of invading pathogens or cellular debris^52–55^. One way the complement-bound materials are eliminated from the blood is via circulating macrophages^53,56^. At the peak synaptic pruning period in the developing visual thalamus, microglia engulf synapses in a complement (C3-CR3)- and neuronal activity-dependent manner^13^. When the critical pruning window is largely over, complement (C1q and C3) activation appears to be down-regulated^12,13,57^. Disruption of complement pruning pathway results in sustained defects on synaptic connectivity^12,13,58,59^, suggesting a fundamental role for the classical complement cascade in brain wiring. Interestingly, a recent study suggested a possible role for complement and microglia in the healthy adult mouse brain involving engram-related memory processes^60^, raising the intriguing question of whether immune pathways critical for synaptic pruning in developing brains contribute to normal synaptic plasticity in the steady-state healthy adult brain. With normal aging, there is a region-specific vulnerability of synapses to loss and dysfunction^61^, and C1q and C3 have been shown to differentially affect age-dependent synaptic vulnerability^57,62^. Together, these studies suggest that the classical complement cascade contributes to synaptic development, maintenance and function throughout the lifespan of an animal.

In AD, complement activation was initially regarded as a secondary event related to peri-plaque neuropathology^63^, as C1q, C3 and C4 are often found up-regulated and localised to neuritic plaques^64^. Moreover, Aβ plaques have been shown to bind and regulate the expression and localisation of complement^65^. However, genetic data suggest that complement may be more than bystanders of AD: among the risk variants for AD are *CLU*, also known as complement lysis inhibitor or *APOJ*, and *CR1*, which encodes for the complement component C3b receptor^66^. Indeed, emerging data in both amyloid- and tau-induced mouse models of AD suggest that the classical complement cascade mediates synapse loss and dysfunction and cognitive impairment^6,8–10,67^. At pre-plaque ages of mouse models of AD (the J20 hAPP and APP/PS1 transgenic), C1q and C3 are reactivated in a brain region–specific manner and appear punctate and localised to synaptic proteins in vulnerable brain regions^6^. In addition, microglia were found to engulf synaptic proteins in a CR3-dependent manner^6^. Importantly, genetic or antibody means of blocking C1q, C3 or CR3 protect synapses from Aβ-induced loss and dysfunction and downstream cognitive impairment^6,8,10^. These findings corroborate those of an earlier study where *C1qa*-deficient mice crossed with the Tg2576 hAPP mouse model resulted in less plaque-related neuronal damage, synapse loss and gliosis compared with *C1qa*-sufficient mice^67^. Similarly, in the Tau-P301S model, unbiased proteomics of hippocampal post-synaptic densities (PSDs) revealed C1q as the most highly up-regulated protein relative to wild-type mice^9^. Injecting anti-C1q functional blocking antibody into the hippocampus of these mice attenuated the loss and microglial engulfment of synaptic proteins^9^. In addition, levels of C1q also positively correlated with levels of phospho-tau in PSDs from the temporal cortex of AD human brains^9^. Genetic deletion of *C3* also rescued neurodegeneration in the Tau-P301S model^10^. Together, these data suggest that the classical complement cascade is reactivated in AD-like brains and mediates synapse loss and dysfunction. Interestingly, inhibiting^68^ or deleting^69^ C3 in one APP mouse model (the J20) resulted in increased plaque-related neurodegeneration whereas C3 deletion in other mouse models (APP/PS1^8^ and PS2APP^10^) resulted in an amelioration of plaque-related neurodegeneration. In a tau-based model, *C3* deletion was protective for neuron loss and brain atrophy^10^. This apparent discrepancy could have stemmed from major differences in the mouse models themselves^8^. However, it is important to note that, despite increased levels of plaques, synapses were still protected from loss and memory was intact in the aged APP/PS1 mice^8^. These studies together suggest that complement is activated in the brain in various contexts to clear what is deemed as ‘debris’ (for example, synapses as well as plaques). Therefore, understanding what on synapses reactivates complement for microglial elimination will be a critical question for the AD field to assess^1^.

## Understanding the molecular determinants of synaptic vulnerability in Alzheimer’s disease

### Apoptosis-like events on synapses in Alzheimer’s disease

Apoptosis, a process of programmed cell death involving caspase-3 activation, has an essential role in triggering the removal of damaged or dying cells by the immune system^55^. Interestingly, Aβ-induced synaptic impairment was ameliorated in caspase-3–deficient rodent models, suggesting that caspase-3 activation is important for Aβ-induced synaptic dysfunction^70^. Caspase-3 activation within hippocampal neurons has been shown to be essential for regulation of spine density and dendrite morphology^71^. Synaptotoxic Aβ species appear to activate local apoptotic cascades, including the cleavage of caspase-3, in synaptosomes and dendrites^14^. Cleaved caspase-3 levels are increased in post-synaptic densities from post-mortem AD human brains^72^ and in hippocampal synaptosomes of pre-plaque Tg2576 hAPP mice at the onset of memory decline and spine loss^15^. These findings collectively suggest that caspase-3 activity contributes to the loss and dysfunction of dendritic spines in AD models and support the notion of focal apoptotic cascades at synapses (that is, ‘synaptosis’)^73,74^. Furthermore, cleaved caspase-3 immunoreactivity was found in spines but not in neuronal soma or pre-synaptic terminals of the Tg2576 hAPP mice^15^, suggesting a potential selective vulnerability of spines in this synaptosis paradigm. Some intriguing questions are whether apoptotic synapses are specifically removed by the immune system and, if so, what mediates this.

### A role for externalised phosphatidylserine at the synapse

A fundamental mechanism employed by the immune system to eliminate damaged or dying cells is the recognition by macrophages of ‘eat me’ and ‘don’t eat me’ signals expressed on the cell surface^55^. PtdSer is a membrane phospholipid that acts as an ‘eat me’ signal on apoptotic cell surfaces^55^. PtdSer is normally asymmetrically localised to the inner leaflet of the plasma membrane, but as cells undergo apoptosis, PtdSer is externalised to the outer leaflet. Cleavage of caspase-3 activates flippases such as ATP11A and ATP11C and inactivates scramblases such as Xkr8, which promote the externalisation and internalisation of PtdSer, respectively^75–77^. ePtdSer on the surface of apoptotic cells then is recognised as an ‘eat me’ signal by macrophages for phagocytosis^55^. Interestingly, ePtdSer has also been proposed to act as a ligand for C1q on apoptotic cells and this binding of C1q to apoptotic cells is inhibited with annexin V, a known PtdSer-binding protein^78^. Recent studies in the developing brain suggest that ePtdSer levels are increased on pre-synaptic compartments during critical periods of circuit refinement^79,80^. Furthermore, ePtdSer-positive neuronal terminals were found within lysosomal compartments of microglia and this localisation was ameliorated in *C1qa* knockout mice^79^. These data suggest a potential role for ePtdSer on synapses as a molecular target of C1q deposition and subsequent microglial engulfment. In the Tg2576 hAPP mouse model of AD, there was an increase of ePtdSer on hippocampal synaptosomes at the onset of hippocampal-dependent memory impairment, synaptic alterations and spine loss^15^. However, whether ePtdSer contributes to synapse loss in AD has yet to be shown.

### Potential links between mitochondrial dysfunction and synaptosis

The activation of caspase-3 on dendritic spines of Tg2576 hAPP mice appears to be dependent on apoptosomes^15^, which are apoptosis-mediating protein complexes formed following the release of cytochrome c from mitochondria^81^. Furthermore, mitochondrial ATP synthase activity, which modulates levels of neuronal PtdSer externalisation^82^, has been shown to be impaired in AD mouse and human brains^83–85^, particularly in synaptic mitochondria^85^. These data suggest a possible link between synaptic mitochondria and synaptosis. In AD human brains, synaptosomes isolated from the temporal cortex have decreased levels of mitochondrial electron transport chain (ETC) complexes I, IV and V, along with an increased level of complement proteins in the same synaptosomes, relative to healthy control subjects^86^. Accordingly, proteomic analysis of the APP/PS1 transgenic mice showed altered levels of mitochondrial ETC proteins in C1q-associated synaptosomes^87^. It is unclear whether these findings are due to decreased protein expression, decreased localisation of mitochondria within synapses or due to preferential loss of mitochondria-rich synapses. However, reduction in the expression of mitochondrial oxidative phosphorylation genes in AD human brains has been shown at the mRNA level^88^. Furthermore, the activity of PtdSer flippases and scramblases can be modulated by ATP^89–92^, reactive oxygen species (ROS)^93^ and intracellular Ca^2+^ levels^92–95^. Mitochondria are critical for supplying ATP and ROS^96,97^ as well as buffering Ca^2+^ following synaptic activity^97–99^. The expression of mitochondrial Ca^2+^ efflux and influx genes is altered in post-mortem AD human brains^100^; and in hippocampal and cortical neurons from hAPP transgenic mice, the ability of mitochondria to buffer Ca^2+^ is impaired^101,102^. Furthermore, the levels of ROS are increased in synaptic mitochondria^103^ and synaptosomes^104^ of pre-plaque hAPP mice relative to wild-type mice. These studies together raise the question of whether mitochondrial dysfunction leads to synapse loss and, if so, how. Further studies are needed to strengthen the role of synaptic mitochondria in synaptosis as well as potential links between synaptic Ca^2+^, ATP and ROS levels with ePtdSer.

## How apoptotic synapses may be recognised for elimination

Tissue-resident macrophages recognise ‘eat me’ signals, such as ePtdSer, on apoptotic cells to mediate engulfment and clearance using a plethora of receptors^55^. Binding of ePtdSer by these receptors can be direct (for example, T-cell immunoglobulin and mucin domain containing 4, or TIM4) or indirect (for example, TYRO, AXL and MER [TAM] receptor tyrosine kinases and α3β5 and α5β5 integrins), the latter of which require ligands to bridge the interaction between receptor and ePtdSer such as GAS6, PROS1 and milk fat globule-EGF factor 8 protein (MFG-E8)^55,105^.

### Potential role for microglial TREM2 in synapse elimination

Of particular interest is TREM2, which has been shown to mediate the clearance of apoptotic cells by macrophages in the brain^106–109^. Genome-wide association studies identified mutations in *TREM2*, such as the R47H loss-of-function variant^110^, as significantly altering the risk for developing AD^111,112^. One mechanism proposed for TREM2 is to act as an immune sensor to detect damage^109,113^. Lipids that accumulate after tissue damage or become externalised on apoptotic cells, such as ePtdSer on neuronal membranes, have been shown to activate TREM2 signalling^108,114,115^. In line with this, multiple studies in AD mouse models suggest that microglia with dysfunctional TREM2 are unable to sense Aβ plaques and thus fail to form a putative protective barrier around plaques^114–120^. TREM2 has also been suggested to be a critical determinant of lipid metabolism in macrophages as well as microglial cell survival^115,121^. In particular, functional knockouts of *Trem2* lead to the inability of microglia to adopt reactive phenotypes (the disease-associated microglia, or DAM)^120–124^. Hence, proper TREM2 signalling may become even more crucial for brain health and homeostasis with aging. An intriguing idea is whether with aging, when the need to clear complement (C1q)-associated synapses increases^57^, aged microglia with decreased lipid metabolic and phagocytic capacity^125^ are unable to efficiently sense or clear what the brain regards as debris.

Loss-of-function mutations in *TREM2* or *DAP12*, an adaptor protein for TREM2 signalling, underlie the Nasu–Hakola disease, in which patients display progressive presenile dementia^126,127^. These findings suggest that TREM2 may have an important role in the maturation and maintenance of synaptic function and connectivity. Indeed, genetic deletion of *Trem2* leads to increased synaptic density and enhanced excitatory neurotransmission in the developing mouse hippocampus^39^, and mice expressing mutations in DAP12 display impaired synaptic maturation^128^. Emerging data further suggest a role for TREM2 in microglia-mediated synapse elimination. Culturing neurons with microglia from *Trem2*-deficient mice prevented synapse loss compared with microglia from wild-type mice^79^. Introducing the humanised R47H variant of *TREM2* into the TauP301S AD mouse model ameliorated C1q deposition on synapses and synaptic localisation within microglia compared with TauP301S mice with the *TREM2* common variant^11^. A similar decrease of synaptic markers within microglial phagolysosomes was displayed in AD post-mortem human brains harbouring the R47H and R62H variants of *TREM2* versus common variants^11^. This apparent neuroprotective role of the R47H or R62H variants, at first glance, does not concur with human genetics^111,112^. However, it may be in line with previous studies suggesting TREM2 as a critical immune sensor for damage and the R47H variant impairing this ability to sense^113^. Akin to what has been shown for the role of classical complement cascade in Aβ-induced synaptic loss versus plaque deposition^6,8^, whether TREM2 is beneficial versus detrimental may depend on the local milieu and the precise insult (that is, the identified ‘damage’ to be cleared)^129^. Future studies, including behaviour and long-term effects on cognitive function, are needed to elucidate the roles of TREM2 in synaptic and cognitive health. Furthermore, whether ePtdSer or other damage-associated lipids contribute to TREM2-mediated synapse elimination in AD and whether this involves the complement reactivation in microglia are unclear.

### Astrocytic MFG-E8 as a potential phosphatidylserine interactor

Astrocytes are intimately associated with synapses, physically^130–133^ and functionally^134^, where they maintain synaptic homeostasis^135^. They have been shown to mediate synapse formation and maturation^136–139^ as well as elimination^40,41,140–142^. Recent data in the developing visual thalamus suggest that astrocytes can mediate synapse loss by direct engulfment of synapses via MERTK and MEGF10^40,142^ and by modulating microglial engulfment of synapses via secretion of IL-33^41^. Interestingly, astrocytes in a given region are highly specialised to meet the demands of the neurons and synapses^132^. This raises the questions of whether and how astrocytes contribute to region-specific synapse vulnerability in disease.

In the peripheral immune system, MFG-E8 has been identified as a bifunctional molecular linker of apoptotic cells to phagocytes^143^; that is, MFG-E8 binds simultaneously to ePtdSer and α5β3 or α5β5 receptors via a C2 domain and RGD motif, respectively^144,145^. *In vitro*, treating with annexin V or cyclical arginine-glycine-aspartic acid (cRGD) integrin-binding motif (which inhibit ePtdSer–MFG-E8 and MFG-E8–receptor interactions, respectively) prevents Aβ-induced engulfment of neurons by microglia^146,147^. *In vivo*, genetic deletion of *Mfge8* reduces lipopolysaccharide-induced neuronal loss in the striatum^148^. Furthermore, tau-laden neurons cultured from P301S-tau mice externalise PtdSer and subsequently are engulfed by microglia and this can be prevented by cRGD^149^. Although these studies have focused on microglial MFG-E8, MFG-E8 appears to be enriched in astrocytes in the brain^150–153^, unlike in the periphery, where MFG-E8 is expressed by tissue-resident macrophages^55,154,155^. In *Drosophila* models, MFG-E8 is involved in the engulfment of dendrites, which display ePtdSer during developmental pruning or upon laser injury^156^. These data together raise the possibility of whether astrocytic MFG-E8 mediates cross-talk with microglia to facilitate synaptic engulfment.

### Potential cross-talk between microglia and astrocytes in mediating synaptosis

Both microglia and astrocytes may be required for complement-mediated synapse loss. In the brain, microglia are a major cellular source of C1q^6,157^ and astrocytes are of C3^158^. Microglia have been suggested to be responsible for the ‘conversion’ of astrocytes to a reactive ‘A1’ phenotype, where C3 is a key marker, through a few factors, including C1q^159^. Furthermore, blocking this conversion appears neuroprotective in two models of neurodegenerative diseases: Parkinson’s^160^ and amyotrophic lateral sclerosis^161^. However, whether astrocytic C3 is required for synapse loss in AD models needs to be further elucidated. Furthermore, microglia and astrocytes both are equipped with clearance machineries, raising the question of whether these two glia cell types have complementary or redundant roles in mediating synapse loss. For example, PtdSer receptors such as TAM receptor tyrosine kinases TYRO3, AXL and MER are expressed by both microglia and astrocytes^40,55,150,158^. Microglial TAM has been shown to mediate the clearance of apoptotic cells in the subgranular zone of the dentate gyrus and the subventricular zone, which are neurogenic regions in the adult central nervous system^162^. Time-lapse *in vivo* imaging showed microglia and astrocytes having distinct functions in the removal of single neurons that were dying upon two-photon ablation^163^, such that microglia appeared to engulf large cell bodies while astrocytes engulf small diffuse debris. *In vivo* spinal cord imaging revealed an intimate physical interaction of astrocytes and microglia upon injury, and this interaction appears to require complement (C3) signalling^164^. Microglia were also suggested to instruct synaptic pruning by astrocytes in synaptic refinement, potentially via TREM2^165^. Together, these data suggest that cross-talk between microglia and astrocytes have important functional consequences on synaptic health and neuronal function^166^.

In aged and AD brains, the transcriptional profiles of microglia and astrocytes are significantly altered^120,153,158,167–171^. In particular, microglia up-regulate PtdSer receptors such as *Trem2* and *Axl*^120,167–170^, and astrocytic expression of PtdSer-bridging molecules such as *Pros1* and *Mfge8* and receptors such as *Megf10* becomes dysregulated^149,153,158,172^. Some intriguing questions are whether the changes of expression of these molecules involved in PtdSer recognition impair the ability of microglia or astrocytes to effectively respond to damaged synapses and neurons and whether they trigger the aberrant removal of otherwise healthy synapses.

## Conclusions

**Figure 1.**
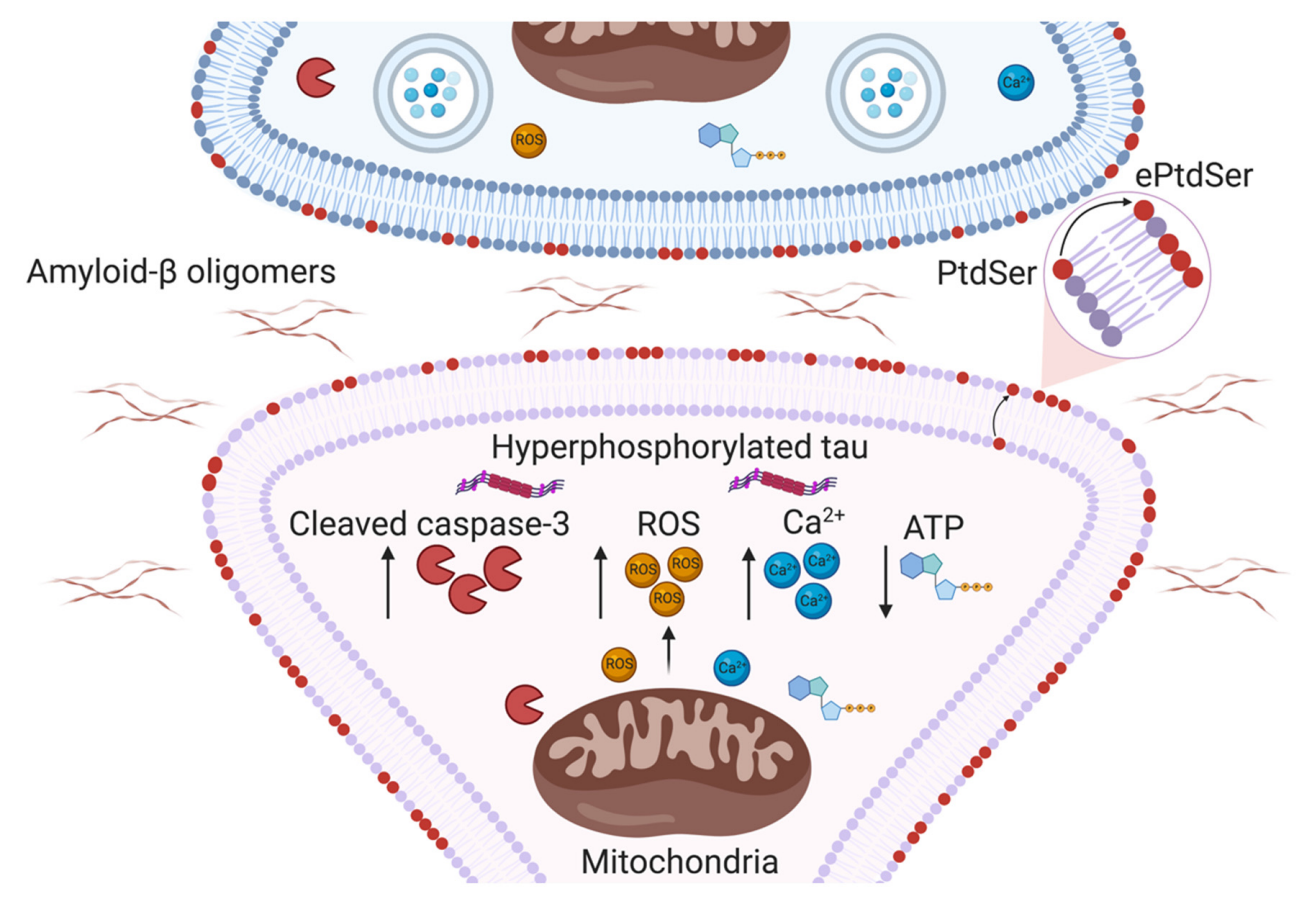
Potential mechanisms leading to synaptic phosphatidylserine externalisation in Alzheimer’s disease. Schematic representation of potential pathways by which oligomeric amyloid-beta and hyperphosphorylated tau may increase the vulnerability of synapses to loss in Alzheimer’s disease. Synaptic mitochondrial dysfunction may lead to a build-up of cleaved caspase-3, reactive oxygen species (ROS) and Ca^2+^, accompanied by a decrease in ATP. These events can modulate the activity of flippases and scramblases, enzymes which regulate the localisation of phosphatidylserine (PtdSer), such that PtdSer is locally externalised to the outer leaflet of synaptic membranes. ePtdSer, externalised phosphatidylserine.

**Figure 2.**
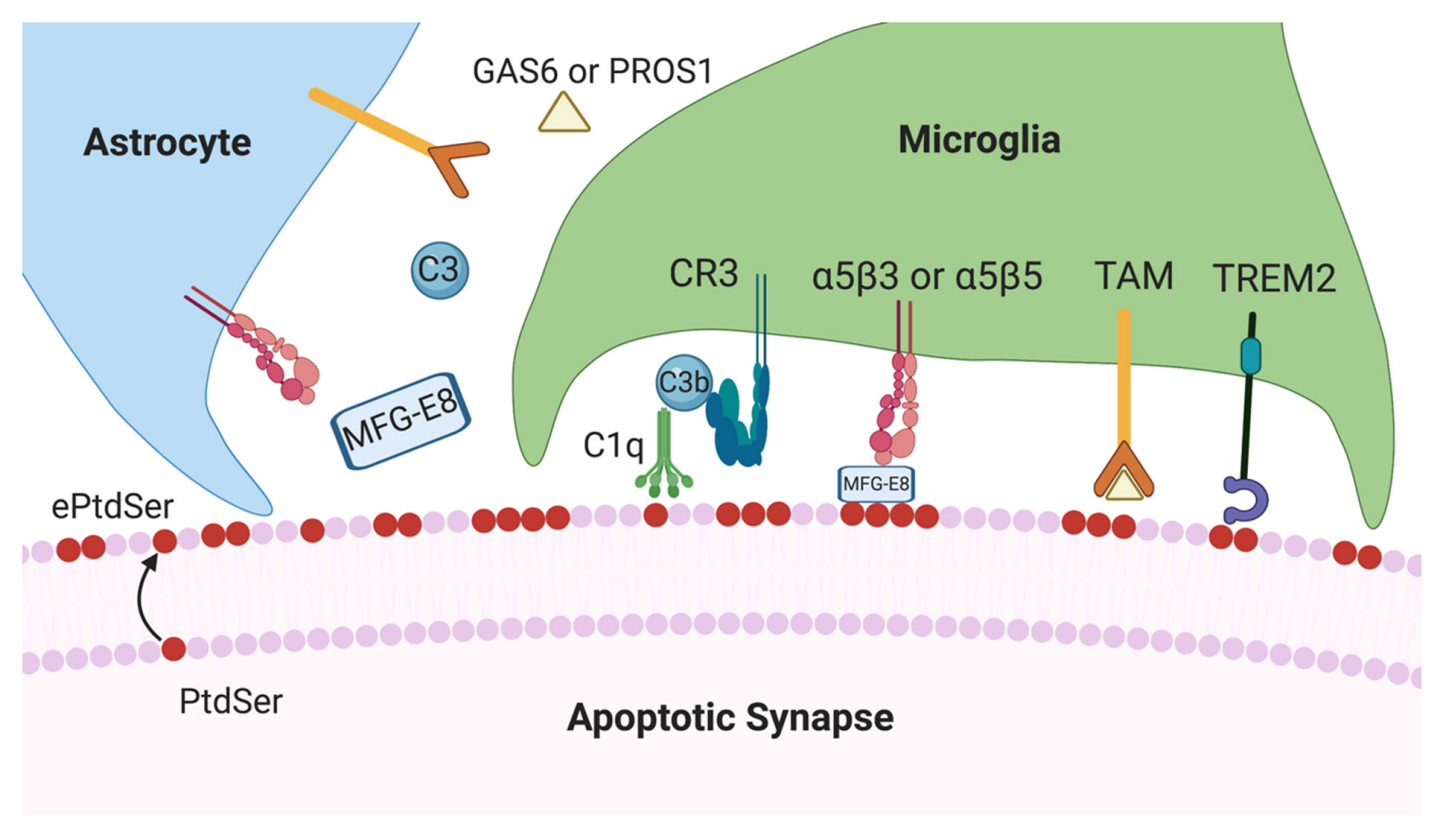
Potential cross-talk between microglia and astrocytes in synapse elimination in Alzheimer’s disease. Schematic representation of proposed glial mechanisms that may mediate the clearance of synapses upon potential externalisation of phosphatidylserine (PtdSer). C1q and C3 proteins secreted by neighbouring microglia and astrocytes, respectively, may mediate engulfment by microglia upon C3b–CR3 interaction. Triggering receptor expressed on myeloid cells 2 (TREM2) may be an important determinant of synapse loss, potentially via recognition of externalised phosphatidylserine (ePtdSer). Astrocytic milk fat globule-EGF factor 8 protein (MFG-E8) may facilitate the interaction between ePtdSer and α5β3 or α5β5 glial phagocytic receptors. Other putative glial PtdSer signalling pathways, such as GAS6/PROS1 and TAM (TYRO, AXL and MER) family of receptors, may also be involved in clearing of synapses with ePtdSer.

Insight into molecular factors mediating region-specific synapse loss will be critical to changing the course of AD. Emerging data suggest that immune mechanisms involving classical complement cascade are critical for synaptic homeostasis, raising the key question of whether certain synapses are targeted for elimination by glia. To this end, recent literature highlights a potential role for ePtdSer in determining synaptic vulnerability. We postulate several pathways, including caspase-3 activation and mitochondrial dysfunction, that may lead to the externalisation of PtdSer on synapses (Figure 1). We then speculate how ePtdSer on synapses may be recognised by microglia or astrocytes (or both) for elimination (Figure 2). In particular, we focus on putative ePtdSer pathways such as TREM2 and MFG-E8. Altogether, we propose that synapses with ePtdSer may be selectively targeted by complement for deposition and subsequent engulfment by glia. However, to the best of our knowledge, no definitive link has been established between ePtdSer, complement and putative PtdSer receptors on glia. Furthermore, whether synaptic mitochondria become dysfunctional and contribute to synapse loss in AD needs further elucidation. As the classical complement cascade and microglia have been implicated in multiple models of neurologic diseases^36^, understanding what makes synapses vulnerable to complement-mediated engulfment and loss will be crucial to resolving neuroimmune interactions critical for brain health.

Importantly, most of these mechanistic insights have been explored in rodent models, which can be a powerful tool to understanding the basic mechanisms of how our brain works. However, it is important to note that striking differences between mice and humans, especially in microglia^170,173,174^, may lead to fundamental differences in complex and chronic age-related neurodegenerative diseases such as AD. Additionally, in Aβ-induced models of AD, synapse loss has been suggested to precede overt plaque deposition^6,175^. However, in patients with AD, when synapses start becoming vulnerable and lost is not fully understood. Recent development of imaging markers that selectively bind to synaptic elements^22^ will be instrumental in better defining the timeline progression of synaptic health in AD.
